# Communication and coordination as drivers of safety behaviours and outcomes in coal-fired power plants

**DOI:** 10.1371/journal.pone.0341341

**Published:** 2026-01-30

**Authors:** Kadir Arifin, Mohamad Xazaquan Mansor Ali, Azlan Abas, Mohammad Lui Juhari

**Affiliations:** 1 Centre for Research in Development, Social and Environment (SEEDS), Faculty of Social Sciences and Humanities, Universiti Kebangsaan Malaysia, UKM Bangi, Malaysia; 2 Occupational Health, Safety and Environment Management (OHSEM) Programme, Faculty of Social Sciences and Humanities, Universiti Kebangsaan Malaysia, UKM Bangi, Malaysia; Bina Nusantara University, INDONESIA

## Abstract

Effective communication and coordination are vital for promoting safety in high-risk industries. While widely regarded as key drivers of safety behaviour, their direct and indirect effects on safety outcomes such as workplace accidents, near misses, fatalities, and lost-time injuries are not well understood, particularly in coal-fired power plants. The study investigates the influence of communication and coordination practices on safety behaviour and their subsequent impact on actual safety outcomes in Malaysian coal-fired power plants. 340 participants from five coal-fired power plants in Peninsular Malaysia participated in a quantitative cross-sectional survey. Stratified random sampling was employed to ensure balanced managerial and non-managerial staff representation. A systematic questionnaire with 60 items was used to gather data. It was divided into four sections: safety outcomes (accidents, near misses, fatalities, and lost-time injuries), safety behaviour dimensions (compliance, involvement, motivation, and knowledge), and demographic data. Five-point Likert scales modified from previously approved instruments were used to measure each issue. Partial Least Squares Structural Equation Modelling (PLS-SEM) in SmartPLS version 3.3.3 was used to analyse the data. The model was evaluated using a two-stage approach to assess formative and reflective constructs. The analysis showed that communication and coordination practices significantly enhanced safety compliance and participation. Among these, safety compliance had a statistically significant negative relationship with adverse safety outcomes. Mediation analysis confirmed that safety compliance played a key role in linking communication and coordination to safety outcomes, indicating that improvements in communication and coordination reduce incidents primarily by fostering better adherence to safety procedures. The results highlight the importance of collaboration and communication as proactive strategies for influencing employee behaviour and lowering unfavourable safety outcomes. One important mediating component surfaced was safety compliance, confirming that management’s coordination and communication efforts work best when they promote constant adherence to safety regulations. In order to foster a culture of safety and operational excellence, organisations in high-risk industries should prioritise establishing transparent, organised communication channels and methodical coordination efforts. These strategies can reduce accidents, injuries, and lost-time incidents while producing quantifiable increases in participation and compliance.

## Introduction

Previous research has highlighted the critical role of communication and coordination in fostering a strong organizational safety culture. Communication is a method of conveying thoughts, feelings, information and knowledge to each other [1] and coordination is vital in informing everyone of progress or any issues arising [2]. Effective communication is the precise and accurate transfer of thoughts, ideas, instructions, or warnings to a specific audience through verbal, written, electronic, or visual means [3]. These practices have been shown to help address safety challenges, prevent workplace hazards, and protect workers from accidents [4–6]. Sharing information about workplace risks and fostering collaboration between managers, supervisors and workers has been linked to improving near-miss reporting, accident prevention and the development of practical safety strategies [7–9]. Strong communication between management and workers is associated with effective risk assessment, increased workplace safety and increased motivation for safe working practices [10,11]. Effective safety communication has also been shown to mitigate the risks of poor communication, such as workplace accidents, reduced productivity, and decreased performance [2,12,13]. As leading indicators of safety performance, communication and coordination provide early warnings of workplace vulnerabilities and facilitate proactive risk management.

The mechanisms through which communication and coordination influence safety outcomes can be further explained through theoretical perspectives such as Social Exchange Theory (SET) and Organizational Learning Theory (OLT). These theories provide a deeper understanding of the mechanisms by which workplace interactions and knowledge-sharing influence safety behaviours and outcomes in high-risk industries. Social Exchange Theory (SET) explains that workplace relationships thrive on mutual reciprocity, where individuals engage in exchanges based on perceived rewards or obligations [14]. In workplace safety, communication and coordination serve as proactive mechanisms that shape employees’ perceptions of organizational support and commitment to safety. When management prioritizes clear safety communication, such as open reporting channels and structured briefings, employees recognize this investment and reciprocate with greater safety compliance and participation. Strong communication fosters trust and commitment, reducing resistance to safety policies and reinforcing compliance as a shared responsibility. Supervisors play a crucial mediating role by providing consistent feedback, reinforcing safety norms, and actively engaging workers, which strengthens motivation and enhances overall workplace safety culture.

Organizational Learning Theory (OLT) highlights the importance of continuous learning and adaptation in enhancing workplace performance [15]. In the context of workplace safety, communication and coordination drive organizational learning by promoting knowledge-sharing, proactive risk management, and systematic safety improvements. Effective communication ensures that critical safety information, lessons from past incidents, and best practices are disseminated across all levels, reinforcing safety procedures. Coordination fosters experience-based learning, enabling teams to refine protocols through post-incident reviews, near-miss analyses, and cross-functional collaboration. Organizations that prioritize structured coordination mechanisms, such as safety committees and interdepartmental planning, develop adaptive safety strategies, ensuring that safety practices evolve in response to real-world challenges.

Despite these findings, the exact mechanisms by which communication and coordination influence safety outcomes remain unclear. Research suggests that their effects are mediated by safety behaviour, which serves as a performance indicator of the effectiveness of communication and coordination [16–18]. However, further research is needed to validate its role in improving safety outcomes, particularly in high-risk industries such as coal-fired power plants. These power plants, which generate a significant portion of Malaysia’s electricity, face unique challenges, including operational risks, costly accidents and power supply disruptions [19–21]. Coal-fired power plants contribute to various environmental and occupational hazards, including the emission of trace elements like selenium, which has been extensively studied for its environmental migration and control policies [22]. Effective communication and coordination are critical for managing these risks and ensuring that safety procedures, legal requirements, and mitigation measures are implemented consistently at all organizational levels.

Effective workplace communication is essential for maintaining situational awareness and ensuring safe operations, particularly in high-risk industries such as coal mining. Research has shown that verbal communication is often preferred in these environments, while discrepancies in written and electronic communication may hinder effective information flow [23]. However, research has shown that mine emergency communication systems often lack standardization, leading to delays in hazard detection and risk mitigation [24]. Similar challenges may exist in other high-risk industries, where incompatible communication networks limit coordination during safety-critical situations. Current studies do not sufficiently address the question of how communication and coordination in such complex environments can specifically mitigate risks and improve safety behaviour.

By integrating SET and OLT, the study highlights that communication and coordination influence safety behaviour. Effective communication fosters trust and engagement, while coordination ensures that safety lessons are continuously internalized and implemented. Together, these mechanisms contribute to enhanced safety compliance, proactive participation, and ultimately, reduced workplace accidents and injuries. This study examines the relationship between communication, coordination, safety behaviour and safety outcomes in Malaysian coal-fired power plants. Coal-fired power plants in Malaysia are characterized by multilingual workforces, hierarchical management, and reliance on subcontractors, which create communication barriers and coordination complexities. These contextual features make safety communication and coordination uniquely critical to achieving compliance and minimizing operational risk. By examining the interplay of these factors, the study aims to provide actionable insights to improve workplace safety, enhance team performance and ensure operational safety in high-risk situations, thus contributing to a deeper understanding of how effective communication and coordination can create a safer and more efficient working environment.

### Communication and coordination as proactive measures

Communication involves the exchange of ideas, knowledge, and information, while coordination ensures that progress and issues are effectively communicated to all relevant parties [1,2]. Both are critical components of a robust safety culture that influence an organization’s safety climate and leadership [5]. Effective communication and coordination enable organizations to proactively address workplace hazards, reduce injuries and promote a safer environment through formal and informal strategies [25,26].

Management’s leadership in communicating safety expectations play a crucial role in influencing worker behaviour and strengthening organizational safety culture. Management with effective communication is critical to workplace safety as it ensures the clear communication of the organization’s vision, objectives, and safety policies [27,28]. The combination of written and verbal methods makes safety rules, procedures, and policies easy to understand and keeps them up to date [29,30]. Regular communication such as meetings and direct feedback on safety performance, increases safety awareness and helps to address unsafe behaviours that can lead to accidents [31,32]. Frequent, open communication between managers, supervisors and workers builds trust and ensures that safety information is shared effectively throughout the organization. Management patrols that emphasize direct interaction and constructive feedback further improve safety outcomes by fostering a proactive environment in which safety concerns are promptly addressed [5,33]. Research supports this claim, as Naji et al. [4] emphasize that clear communication from management enhances safety compliance. Similarly, Kines et al. [5] found that management-led safety communication led to a reduction in incidents within high-risk industries. Moreover, Lee et al. [27] highlights that strong management communication fosters trust and encourages worker engagement in safety policies, further reinforcing a culture of safety in the workplace.

Effective safety reporting systems facilitate hazard identification, incident documentation, and corrective action implementation, ultimately reducing future accidents. Where reporting and feedback mechanisms are critical to maintaining a positive safety climate. They focus on identifying the causes of errors and accidents rather than apportioning blame [34,35]. A well-structured reporting system ensures confidentiality and anonymity and encourages workers to report hazards without fear of retaliation [36,37]. Prompt responses to reported issues build trust and encourage collaboration. Sharing the results of safety reports encourages collaborative problem solving and motivates workers to actively participate in hazard identification [34,38]. An open communication culture led by management demonstrates commitment to safety and strengthens worker trust and cooperation [39,40]. Reason [35] states that safety reporting minimizes risks by identifying systemic vulnerabilities, while Hopkin [36] found that confidential reporting mechanisms improve the overall safety climate. Additionally, Havinga et al. [34] highlight that organizations with strong reporting cultures tend to have lower accident rates.

Supervisors play a key role in communicating safety priorities and influencing worker behaviour by acting as intermediaries between workers and management, reinforcing safety policies, and ensuring compliance. Proactive monitoring and feedback by supervisors emphasize the importance of safety over production goals, thereby improving work performance and reducing risk [5,33]. Constructive problem solving between supervisors and workers fosters a positive communication climate that leads to better engagement and adherence to safety protocols [11,33]. Supervisors who emphasize social support and priorities safety behaviour contribute to improved safety systems and a better organizational climate [41]. Sharing responsibilities and clarifying safety expectations increases commitment and reduces the likelihood of accidents [11,42,43]. Research supports the importance of supervisory communication in workplace safety, as Michael et al. [33] found that supervisor-led communication enhances worker adherence to safety protocols. Cigularov et al. [1] demonstrated that supervisors significantly influence workers’ perceptions of safety through regular engagement, while Kessler et al. [7] identified frontline supervisors as key figures in accident prevention.

Coordination is essential for the harmonization of activities, particularly in organizations with diverse objectives, as it ensures that safety measures are effectively integrated into daily operations, thereby reducing task-related risks. Poor coordination can lead to increased risks, especially when tasks are outsourced or multiple stakeholders are involved [2,44]. Planning and scheduling work effectively helps mitigate safety risks, especially for critical or high-risk activities [45]. Standardized safety policies and procedures ensure consistency and reduce the potential for accidents due to non-compliance [46]. Regular monitoring and inspection of tasks ensure that safety plans are effective and that the necessary corrective actions are taken when problems arise [47]. Adherence to safety rules and protocols strengthens collective performance and fosters a shared sense of responsibility and accountability [48]. Research highlights the importance of coordination in workplace safety, as Al Nahyan et al. [2] emphasize that coordination is crucial for hazard control in complex work environments. Okhuysen & Bechky [48] argue that interdepartmental coordination plays a key role in minimizing safety failures, while Nenonen [46] found that poor coordination significantly increases accident risks in outsourced operations.

The study used these four dimensions based on their strong influence on workplace safety performance and their empirical validation in previous research through driven leading indicators found in the literature. While other dimensions can contribute to safety outcomes, they are indirect factors or fall outside the core framework of communication and coordination as defined in ISO 45001:2018. To summarize, the cornerstone of workplace safety depends on effective communication, improved safety reporting, good communication between supervisors and established safety coordination. This approach reduces accidents and injuries and increases trust, commitment, and the overall safety climate. This study emphasizes the importance of these factors in management planning to improve safety in the workplace.

### Effect on safety behaviours

Research shows that unsafe actions significantly increase accident risks, whereas compliance with safety procedures effectively reduces them [46,49,50]. Safety management practices, as antecedents, shape safety outcomes by influencing safety knowledge, motivation, and behaviors critical to minimizing accidents [51–53]. Effective communication and coordination within organizations are crucial factors in improving safety performance [4].

The ability of workers to understand and execute tasks safely, use protective equipment, identify hazards, and implement precautionary measures is essential for workplace safety [18]. Effective communication and coordination will enhance safety knowledge, enabling workers to uphold safety standards and prevent incidents [6,18,54]. Whereas, safety motivation plays a crucial role in encouraging workers to follow safe practices, actively participate in safety activities, and improve overall safety performance [51,55,56]. It involves workers valuing health and safety, prioritizing personal safety, reducing risks, and fostering a collaborative approach to promoting safety practices among colleagues [52,54,57].

Safety compliance refers to adhering to established safety rules and procedures, which is essential for maintaining workplace health and safety [58,59]. It involves workers consistently using safety equipment, following proper procedures, reporting incidents accurately, and ensuring personal protective measures are applied during tasks [60,61]. Safety participation involves proactive behaviors that foster a safety-oriented environment, such as assisting colleagues, promoting safety programs, and providing constructive suggestions, even if these actions do not directly impact personal safety [49,62]. It is demonstrated through efforts like volunteering to enhance workplace safety, encouraging others to follow safety practices, addressing safety issues, and taking action to prevent violations [54,60].

Adherence to safety procedures, supported by effective, strong organizational communication and coordination, significantly reduces the risk of accidents by improving safety knowledge, motivation, participation and compliance. Communication and coordination improve workplace safety by relying on workers’ ability to perform tasks safely, use protective equipment, recognize hazards and comply with safety standards. This motivates workers to prioritize health, reduce risks and promote a cooperative safety culture. In addition, effective communication and coordination increases compliance, the application of protective measures and the reporting of incidents.

In addition, proactive behaviour, such as promoting safety programmed, supporting colleagues and raising safety issues thus improves the safety working environment. Based on the literature, the study expects that communication and coordination practices will positively affect safety knowledge; safety motivation; safety compliance; and safety participation. These behaviours will in turn influence outcomes. Safety knowledge, safety motivation, safety compliance, and safety participation were evaluated as observant leading indicators based on the previous studies.

### Safety outcomes as final goal

Safety outcomes, influenced by various factors and conditions, reflect the effectiveness of organizational safety efforts in reducing accidents and injuries [63,64]. In this study, the authors aim to examine the role of safety communication and coordination within organizations in improving safety performance, thus achieving safety outcomes. Safety outcomes, whether temporary or part of ongoing processes, provide critical insights into the success or failure of safety interventions, even if they do not directly indicate an organization’s overall safety level [65,66]. The primary goal is to minimize incidents such as accidents, fatalities, near misses, and lost time injuries, all of which negatively impact the workplace, underscoring the importance of continuous safety improvement [67,68].

In the electrical utility industry, occupational accidents frequently result in fatalities or severe injuries, such as organ damage from electric shocks [69,70]. However, workers face additional risks beyond electrical hazards [69,71,72]. These accidents are assessed by their frequency and the severity of resulting injuries, ranging from minor harm to temporary or permanent disabilities [8,71]. The impact is further measured through the associated medical costs required for treatment, highlighting the financial burden of workplace injuries and the importance of robust safety measures [73].

Fatal accidents are measured by the number of workplace fatalities and are the focus of investigation and prevention efforts as they highlight the risks associated with working conditions [74–77]. These incidents cause considerable costs due to investigations, administrative processes and legal action [78]. In addition, productivity suffers due to the loss of skilled labor, while the hiring and training of replacements requires a lot of time, effort and financial resources, which further affects the efficiency of the company [79,80].

Near misses are events that have the potential to cause accidents but result in no injuries or only minor property damage [81–83]. They are often caused by unsafe conditions or behaviors and, although they do not result in immediate damage, can still cause significant costs due to necessary repairs, such as maintenance of machinery or restoration of equipment [84–86]. Recognizing and addressing near misses is critical to preventing dangerous incidents as they serve as an early warning system for the underlying safety risks [82,83,87].

The term “work loss” refers to a worker’s inability to work due to pain or injury that results in a loss of productive hours and measured by the frequency of injuries per million hours worked per year [72]. Such injuries incur significant costs to organizations, including wages for replacement workers or overtime pay to maintain production levels [84,85]. In addition, treatment-related absences interrupt productivity and contribute to productivity losses [84]. Further costs arise from operational downtime and the time required for investigations, all of which have a negative impact on production and the overall efficiency of the organization [85,88].

Several studies support an indirect link: management safety practices (communication, training, etc.) improve intermediate outcomes (like safety climate and behaviours), which then reduce accident rates [51,63,89]. The study expects that higher safety compliance and safety participation will correlate with lower accident/injury rates and that compliance and participation will carry the effect of communication/coordination to those outcomes. These indicators provide valuable insights into workplace safety performance and the effectiveness of safety management practices.

### Structural model development

**Fig 1:**
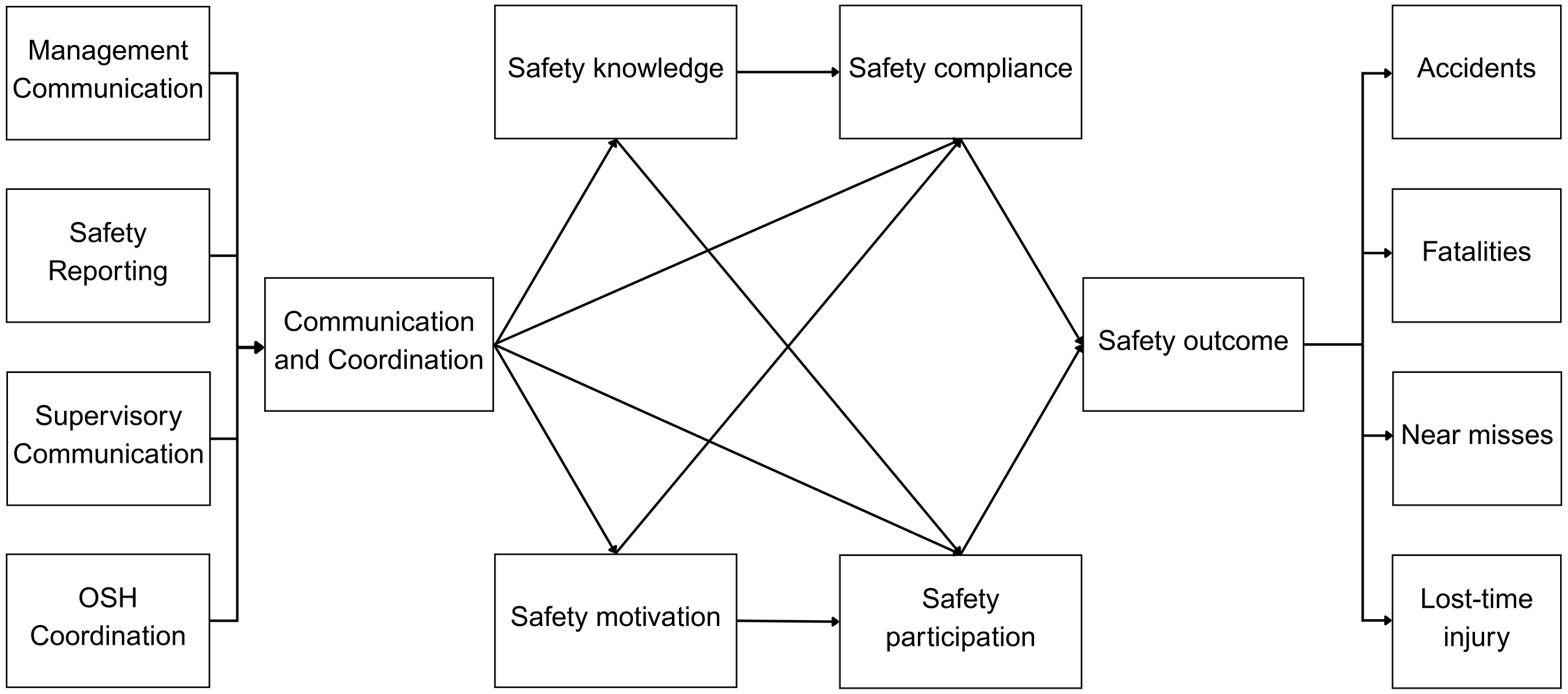
Conceptual relationship model.

The study highlights the importance of communication and coordination in safety on how specific communication styles or frameworks directly or indirectly affect safety behaviors and safety outcomes. By examining communication and coordination as leading indicators, organizations can deepen their understanding of how these factors improve safety behavior and contribute to better safety outcomes. It investigates: (1) whether communication and coordination directly affect workers’ safety behaviors (2) whether safety behaviors mediate the relationship between communication/coordination and safety outcomes such as accidents, near misses, and lost time injuries; and (3) the extent to which these leading indicators serve as foundational mechanisms to influence safety outcomes. The proposed model and structural relationships are illustrated in Fig 1.

## Research methodology

### Questionnaire design

This study employed a structured questionnaire to measure the impact of communication and coordination on safety behavior and safety outcomes. The questionnaire was designed based on an extensive review of relevant literature and validated instruments from previous research as shown in Tables 1, 2 and 3. The development process included expert consultations, pilot testing, and refinement to ensure construct validity and reliability. The questionnaire comprising four sections was used to collect data for this quantitative study: (1) respondent information; (2) communication and coordination (20 questions); (3) safety behaviors (20 questions); and (4) safety outcomes (20 questions). The questionnaire, developed based on a literature review, contained both positively and negatively worded questions to minimize social desirability bias [90,91]. To ensure consistency, all negative responses were reversed when analyzing the data. To further reduce bias, participants were assured confidentiality and anonymity in a cover letter, and no personal data such as names or signatures were collected [92].

**Table 1:** Result of descriptive and reliability analysis.

No	Construct	No. of Item	M (SD)	Cronbach’s alpha (α)
**Communication and coordination (COC)**				
1	Communication (MNC)	5	4.14 (0.49)	0.647
2	Reporting (SFR)	5	4.08 (0.53)	0.727
3	Supervisory (SVC)	5	4.07 (0.49)	0.683
4	Coordination (SCO)	5	4.13 (0.51)	0.712
**Safety behaviours**				
5	Knowledge (SFK)	5	4.37 (0.57)	0.796
6	Motivation (SFM)	5	4.52 (0.54)	0.883
7	Compliance (SFC)	5	4.46 (0.54)	0.780
8	Participation (SFP)	5	4.36 (0.54)	0.717
**Safety outcomes (SFO)**				
9	Accident (OAC)	5	2.05 (0.90)	0.719
10	Fatalities (FAC)	5	2.03 (0.98)	0.775
11	Near misses (NRM)	5	2.02 (0.87)	0.925
12	Lost Time Injury (LTI)	5	2.28 (0.96)	0.943

**Table 2:** Full collinearity testing.

COC	SFK	SFM	SFC	SFP	SFO
1.125	1.544	1.55	2.161	2.112	1.043

**Table 3:** Testing indirect effects of communication and coordination towards safety outcomes.

Relationship	*β*	Std Error	T-Values	P-Values	LL	UL
**COC → SFC → SFO**	−0.088	−0.091	2.91	0.004	−0.152	−0.032

Note: 5000 samples of bootstrapping with 95% confidence interval.

A five-point Likert scale ranging from 1, very low to 5, very high to rate safety conditions as suggested by Guo et al. [93] to evaluate communication and coordination, and safety outcomes. Following Neal et al. [52], a five-point Likert scale was used ranging from 1, strongly disagree to 5, strongly agree to evaluate safety behaviors. The Cronbach’s coefficient is used to assess the reliability of scales, meaning that a variable or a set of indicators of a latent construct are internally consistent in their measurements [94]. Items were adapted from validated scales and refined through expert review and pilot testing. The measurement model was verified using PLS-SEM’s two-stage approach following Becker et al. [95], Sarstedt et al. [96] and Hair et al. [97], which provides convergent validity based on Average Variance Extracted (AVE) and discriminant validity based on Heterotrait-Monotrait (HTMT) ratio of correlations which equivalent to CFA.

### Sampling approach and data collection

The research focused on workers at five coal-fired power plants in Peninsular Malaysia: Tanjung Bin Power Plant, Johor; Janaelektrik Sultan Salahuddin Abdul Aziz, Selangor; Jimah Power Plant, Negeri Sembilan; Stesen Janakuasa Tuanku Muhriz, Negeri Sembilan; and Stesen Janakuasa Sultan Azlan Shah, Perak. The respondents for this research were local workers, comprising 748 executives and 2,132 non-executives. According to Krejcie & Morgan [98] and Hair et al. [97], a minimum sample size of 300 is sufficient for models containing up to 10 constructs and medium effect sizes (0.15). The final sample of 340 respondents thus provides adequate statistical power (β = 0.80) for detecting significant structural relationships needed for PLS-SEM analysis. Therefore, the sample for this research was set to 88 respondents representing the executives and 252 respondents representing the non-executives using stratified random sampling.

The study involving human participants was reviewed and approved by the Universiti Kebangsaan Malaysia and the Department of Occupational Safety and Health Malaysia as a regulatory body. All procedures followed the 1964 Helsinki Declaration and its subsequent changes, as well as the institutional research committee’s ethical guidelines. Written informed consent was obtained from all participants prior to their inclusion in the study. Participants were provided with detailed information about the study’s objectives, procedures, and benefits. They were informed that participation was voluntary and that they could withdraw at any time. The survey was carried out during the COVID-19 epidemic, more precisely while the government was enforcing the Movement Control Order (MCO). The period of data collection was October 2021–January 2022. The researchers emailed the questionnaire after it had been approved by each participating power plant’s health and safety manager. The researchers used a validated list of employees’ registered email addresses that the individual organizations supplied to guarantee accurate and direct distribution to every respondent. Participants were provided with detailed information about the study’s objectives, procedures, and benefits. They were informed that participation was voluntary and that they could withdraw at any time.

### Analytical methods

The demographic, descriptive and reliability variables were initially analyzed using SPSS version 26. The pat analysis was performed according to the guidelines of Ramayah et al. [99] and Hair et al. [100] using SmartPLS version 3.3.3 for partial least squares (PLS). The model was evaluated using the two-stage approach based on guidelines [96], which included two higher-order constructs (HOCs): communication and coordination practices and safety outcomes. Following Becker et al. [95], the communication and coordination practices HOC construct was developed as a formative-formative model, suited for assessing performance in complex constructs with numerous indicators. In contrast, the safety outcomes construct was designed as a reflective-reflective model, measured reflectively with distinct yet correlated indicators.

The reflective construct model validity and reliability were assessed using Cronbach’s alpha > 0.6, loading values ≥ 0.5, average variance extracted (AVE) ≥ 0.5 and composite reliability (CR) ≥ 0.7. For the formative construct, loading values with a T-value ≥ 1.964, an outer loading ≥ 0.5 and a variance inflation factor (VIF) below 5.0 were used in the assessment of validity and reliability. The model developed included higher-order formative components for which internal consistency reliability and convergent and discriminant validity were not assessed, as strong correlations between items in formative constructs are unnecessary [101].

## Result

### Demographic distribution

The respondents included 88 executives (25.88%) and 252 non-executives (74.12%). Among executives, the largest educational group consisted of bachelor’s degree holders (63 respondents, 18.53%), while among non-executives, diploma holders were the majority (213 respondents, 62.95%). Overall, most respondents held a diploma (220 respondents, 64.71%), while doctorate holders were the smallest group (2 respondents, 0.59%). In terms of gender, the executive group comprised 83 men (24.41%) and 5 women (1.47%), while the non-executive group comprised 251 men (73.82%) and 1 woman (0.29%). Most respondents were between 31 and 40 years old, 44 senior executives (12.94%) and 154 non-executives (45.29%) made up a total of 198 respondents (58.24%). Respondents over the age of 51 formed the smallest age group with 5 executives (1.47%) and 15 non-executives (4.41%).

### Descriptive analysis

The mean values (M), the standard deviation (SD) and the reliability result are shown in Table 1. The reliability of the measured values is estimated using a reliability coefficient called Cronbach’s alpha (α) for internal consistency [102]. A α value of 0.7 or higher is considered satisfactory as it demonstrates a strong internal consistency of established scales in basic research [103]. However, a α score of 0.6 or higher is also considered significant [97]. In this study, all constructs were found to have a α value between 0.647 and 0.943, so the results of Cronbach’s alpha (α) for all constructs indicate high internal consistency and good agreement with the respondents.

The average mean score shows that communication and coordination in coal-fired power plants is high in management communication (MNC), safety reporting (SFR), supervisory communication (SVC) and safety coordination (SCO) as the mean scores ranged from 3.41 to 4.20. Respondents rated safety behaviour in terms of safety knowledge (SFK), safety motivation (SFM), safety compliance (SFC) and safety participation (SFP) as very high, with a mean score of more than 4.21. In contrast, the safety outcomes consisting of occupational accidents (OAC), fatal accidents (FAC), near misses (NRM) and lost time injuries (LTI), were low with a mean score between 1.81 and 2.60.

### Path analysis

The data were collected from the same source and common method bias (CMB) was performed to test the data for bias, as shown in Table 2. All variables are regressed against a random dependent variable to determine if there is a bias from single source data. If the VIF of the within model is less than 3.3, then there is no bias from the source data. The analysis revealed an inner model VIF of less than 3.3, so single source bias was not a serious problem with this data. Furthermore, the study used self-reported thus addressing the risk of standard method bias by using statistical and procedural remedies. Respondents were guaranteed anonymity throughout the procedure, and the researchers thoughtfully crafted the study’s question phrasing to reduce social desirability bias and evaluation anxiety. The researchers perform Harman’s single-factor test statistically. According to the research, the first component only explained 18.48% of the variation, significantly less than the 50% criterion for significant standard method bias that is generally acknowledged [104]. This implies that common method bias is not a significant issue in this research.

#### Measurement model.

The communication and coordination model were evaluated using a separate two-stage approach to validity and reliability. Communication and coordination were modelled as formative–formative HOCs due to the indicators are conceptually distinct and contribute collectively to the construct, consistent with Becker et al. [95] and Sarstedt [105] suggestion. Each subconstruct namely, management communication, reporting, supervisory and coordination contributes uniquely to the overall construct without assuming internal correlation. The first-stage measurement analysis for the formative constructs revealed that all external loadings were above 0.5, although most of the items did not have a significant external weight. CC3, CC6, CC8 and CC19 were dropped due to insignificant outer weight values and loading values of less than 0.5, as shown in Annex 1. However, the number of dropped items does not exceed 20 per cent of the total items and is still acceptable. The VIF values of less than 3.3 show that there are no collinearity problems. The results show that the first order of the formative construct was valid and reliable. The second stage analysis for the formative construct showed that the outer loading value is more than 0.8 even though there is no significant outer weigh value. In addition, the second stage of the formative construct shows no collinearity problem as the VIF value is less than 3.3.

**Annex 1. Measurement of the formative-formative constructs in high-order construct.**

The measurement analysis of the HOC reflective constructs of safety outcomes through the first and second stages are shown in Annex 2. All constructs found to have loadings greater than 0.7, except for item NM5 in the first stage. However, it is acceptable as only one loading falls below 0.708, consistent with the guidelines by Hair et al. [100]. Confirming the reliability and validity criteria, other measurement show that the AVE values exceed 0.5, Cronbach’s alpha values are above 0.6, and CR values are greater than 0.7. Therefore, the validity test result for formative-formative HOC and reflective-reflective HOC is valid and reliable.

**Annex 2. Measurement of the reflective-reflective constructs in high-order construct.**

The reflective construct measurement for the low-order constructs (LOC) of SFK, SFM, SFC, and SFP valid and reliable base on the AVE values exceeding 0.5, Cronbach’s alpha values above 0.6, and CR values greater than 0.7 as shown in Annex 3.

**Annex 3. Measurement of the reflective construct in low-order construct.**

The heterotrait-monotrait (HTMT) ratio of the correlation analysis for the reflective constructs shows values below 0.9 for all latent variables, which confirms discriminant validity, as shown in Annex 4. Consequently, the overall model of communication and coordination is considered valid and reliable after analyzing it with a two-stages approach.

**Annex 4. Discriminant validity (HTMT).**

#### Structural model.

**Fig 2:**
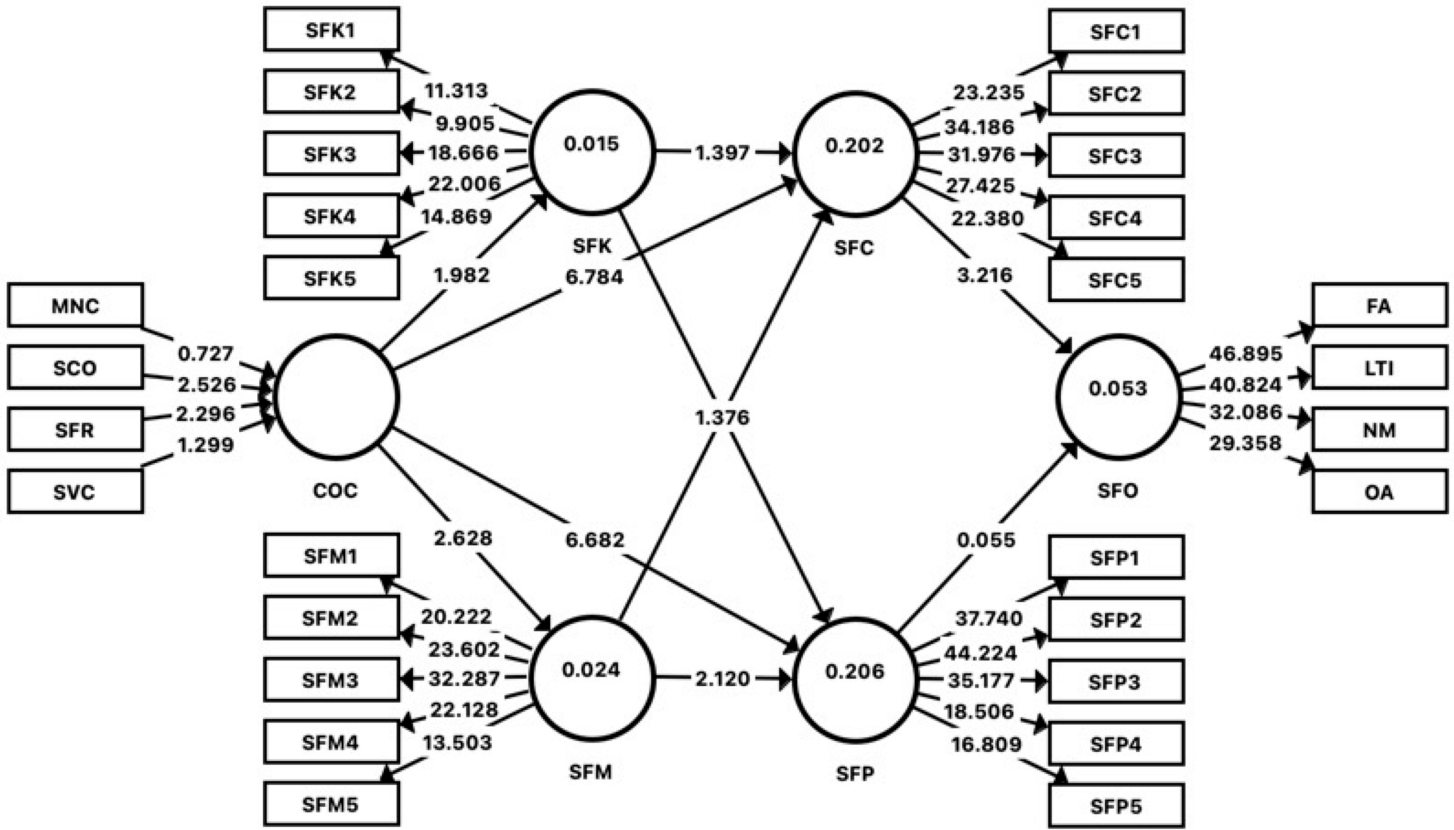
Bootstrapping results in the model for the second-order constructs.

Multivariate skewness and kurtosis were assessed following guidelines from Hair et al. [97] and Cain et al. [106] to evaluate multivariate normality. Mardia’s coefficients for multivariate skewness (β = 7.182, p > 0.01) and kurtosis (β = 55.467, p > 0.01) indicated a violation of multivariate normality assumptions. This justified the use of Partial Least Squares Structural Equation Modelling (PLS-SEM), which is robust to non-normal data distributions. Relationships among the core constructs were examined using PLS-SEM with bootstrapping of 5,000 samples, following recommendations by Ramayah et al. [99] and Hair et al. [100], as shown in Annex 5 and illustrated in Fig 2.

**Annex 5. Testing direct effects of communication and coordination towards safety outcomes.**

Annex 5 summarizes the results, including standardized beta coefficients, standard errors, t-values, p-values, R^2^ values, f^2^ effect sizes, and 95% bias-corrected and accelerated (BCa) confidence intervals (CI 2.5% – CI 97.5%). The inclusion of CIs provides additional insight into the precision and reliability of the estimated effects: if a confidence interval does not include zero, the corresponding path is considered statistically significant at the 5% level. Out of ten direct relationships tested, four were statistically significant:

1. The path COC → SFC (β = 0.388, t = 6.784, p < 0.001, CI [0.273, 0.490]) demonstrates a strong, positive, and statistically significant effect of communication and coordination on safety compliance. Since the CI range does not include zero, this result is statistically robust. The effect size is medium (f^2^ = 0.184), and R^2^ = 0.202 indicates that 20.2% of the variance in safety compliance is explained by communication and coordination.
2. Similarly, COC → SFP (β = 0.375, t = 6.682, p < 0.001, CI [0.266, 0.482]) shows a significant and positive influence on safety participation, with a medium effect size (f^2^ = 0.173) and R^2^ = 0.206.
3. COC → SFM (β = 0.156, t = 2.628, p = 0.009, CI [0.050, 0.264]) also emerged as significant, albeit with a smaller effect size (f^2^ = 0.025). The confidence interval again excludes zero, reinforcing the result’s statistical validity.
4. The relationship SFC → SFO (β = −0.227, t = 3.216, p = 0.001, CI [−0.372, −0.093]) is statistically significant and indicates a negative relationship, suggesting that higher safety compliance is associated with fewer safety-related incidents. The effect size is small (f^2^ = 0.025), but the result remains meaningful for policy and practice.

For the remaining paths, the confidence intervals include zero, indicating that those relationships were not statistically significant despite some having non-zero beta estimates. For example, SFM → SFP (β = 0.155, p = 0.034) has a CI [0.011, 0.291] that narrowly excludes zero, making it borderline significant and warranting cautious interpretation.

Table 3 shows the results of analyzing the indirect effect of communication and coordination (COC) on safety outcomes (SFO) through safety compliance (SFC). The standardized path coefficient for the indirect effect is −0.088, indicating a negative indirect relationship between communication and coordination (COC) and safety outcomes (SFO) through safety compliance (SFC). This suggests that improved communication and coordination may indirectly lead to a reduction in adverse safety outcomes via safety compliance. The indirect effect is statistically significant at a 5% significance level, confirming the existence of an indirect relationship (t = 2.91, p < 0.05). The lower limit (LL) and upper limit (UL) of the confidence interval range from −0.152 to −0.032. Since the interval does not include zero, the result is statistically significant, which further supports the existence of an indirect effect [107,108].

Additionally, the predictive capability of the model was assessed using PLS-Predict with a 10-fold procedure applied to a bridging sample, which generated predictions at both the construct and item levels, following the approach outlined by Shmueli et al. [109]. According to their guidelines, predictive power is considered strong if all item differences (PLS-LM) are lower, moderate if most are lower, low if only a minority are lower, and unconfirmed if all are higher. In this study, 63% of the prediction errors in the PLS model were lower compared to the LM model, indicating a moderate level of predictive power.

## Discussion

This research addresses a significant topic in the fields of safety and social sciences, highlighting communication and coordination as proactive leading indicators that influence safety behaviour, which serves as an observational leading indicator and ultimately affects safety outcomes as a lagging indicator. Much of the existing literature examines the impact of safety management practices on safety performance rather than directly on safety outcomes due to the complicated nature of this relationship. The main objective of this study is to evaluate and discuss the empirical model developed to investigate the relationships between leading indicators and safety outcomes. These findings align with prior research suggesting that strong organizational communication enhances workplace safety and fosters a culture of compliance [4,5].

The study on communication and coordination practices in coal-fired power plants in Malaysia emphasizes the central role of communication and coordination (COC) as key factors for safety participation (SFP) and safety compliance (SFC), both of which show a medium effect size in the analysis conducted. The research results show that effective communication and coordination can positively influence safety performance, especially safety compliance, which confirms the findings of by Ashour & Hassan [110] and Ajmal et al. [111]. Furthermore, this study agrees that safety management practices, especially communication, predict safety participation, supporting the study by Ashour & Hassan [112] and Keffane [113]. The relationship supports that the level of safety performance is dependent on management’s emphasis on regular and direct communication throughout the organization. Through communication and coordination, management can control safety issues and ensure that workers are protected from potential hazards and accidents. Showing that consistent and clear communication, coupled with effective coordination, is crucial in shaping safety behaviours by ensuring workers understand protocols, feel valued in decision-making processes, and are actively engaged in maintaining a safe and compliant workplace culture [114,115]. These results are consistent with Griffin & Neal [116] that highlighted that effective communication strategies facilitate the internalization of safety policies and procedures, enhancing compliance and participation.

In contrast, safety knowledge (SFK) and safety motivation (SFM) show weaker or non-significant direct effects, emphasizing that enhancing communication and coordination may be more effective in driving safety behaviours. The weaker or non-significant effects of safety knowledge (β = 0.121, p = 0.048) and safety motivation (β = 0.156, p < 0.05) suggest that while these factors contribute to safety behaviour, they may not be as strong as direct communication and coordination practices. This finding suggests that safety leadership and structured communication channels play a more immediate role in driving safety compliance and participation than intrinsic safety knowledge or motivation. Therefore, the effectiveness of implementing communication and coordination can be measured by examining its impact on safety compliance and safety participation.

The key finding of this study highlights the mediating role of safety compliance in the relationship between communication and coordination practices and safety outcomes. The results indicate that the effectiveness of communication and coordination (leading indicators) relies on their influence on safety compliance (observational leading indicator) before contributing to safety outcomes (lagging indicators). A negative correlation was observed between safety compliance and safety outcomes, demonstrating that improved compliance reduces adverse safety events. Similarly, safety outcomes were inversely associated with communication and coordination practices, indicating that better implementation and effectiveness of these practices enhance safety compliance, ultimately lowering occupational accidents, fatalities, near misses, and lost time injuries. These findings align with prior research [111,117,118], supporting the idea that safety management practices mitigate workplace accidents and injuries through the mediating effect of safety compliance. This underscores the intricate nature of safety dynamics and emphasizes the importance of comprehensive strategies focusing on communication, coordination, and motivation to enhance workplace safety outcomes.

The study’s findings reinforce the importance of leadership-driven communication strategies in enhancing workplace safety. Prior studies by Lee et al. [27] and Reese [30] support the notion that clear, proactive communication from management significantly improves safety climate and reduces workplace incidents. Moreover, this research confirms that open safety reporting mechanisms, where workers feel safe to report hazards without fear of retaliation, lead to improved compliance and proactive safety behaviors [35,36]. The results also support the work of Kessler et al. [7] and Cigularov et al. [1], who found that supervisors play a crucial role in reinforcing safety behaviours through direct engagement and feedback. In the context of this study, the significant effect of supervisory communication on safety compliance highlights that frontline supervisors serve as key mediators in ensuring that safety policies are effectively translated into daily practices. Regarding coordination, this study corroborates findings by Al Nahyan et al. [2] and Okhuysen & Bechky [48], which emphasized that well-structured safety coordination leads to more effective hazard control, particularly in high-risk industries. The study further confirms that standardized safety policies and clear safety roles among workers help minimize risks associated with outsourced or high-hazard tasks [46].

Limitation of this study is that it focuses on communication and coordination practices as primary leading indicators of safety. Future research should examine a broader range of safety management practices, including managerial commitment, worker involvement, hazard identification and control, training, education, and continuous improvement as described in the ANSI Z10 and ISO 45001 standards. Researchers should consider integrating these practices into a unified construct to help safety professionals design, implement, and advocate for effective safety management systems in the workplace. However, it is important to recognize the limitations of the PLS-SEM, which can only consider two levels of constructs. The inclusion of multiple leading indicators in a single construct can increase the variance inflation factors (IVF) of the indicators, which poses a challenge for data analysis [97,99,101]. Another limitation of this study is that it focuses on observable leading indicators of safety behaviour and safety performance as mediators of safety outcomes. Future research should examine other factors that influence the effectiveness of communication and coordination efforts. These factors should be consistent with the definition of observable leading indicators which provide insights into system dynamics, including questions about ongoing activities, capabilities, skills, motivation, routines, practices, and the overall potential of the organization to ensure safety [119]. These indicators are used to assess the effectiveness of the safety programmes and activities implemented by the organization. Lastly, the use of self-reported data may involve perceptual bias despite anonymity. Additionally, the cross-sectional design restricts causal interpretation; future longitudinal studies are recommended to confirm temporal causality.

In addition, future research should examine positive outcomes that enhance organizational performance through improved workplace safety and health. While this study focuses primarily on reducing negative outcomes such as workplace accidents, fatalities, near misses and lost time injuries, as highlighted by Christian et al. [51], Vinodkumar & Bhasi [57] and Clarke [53], it is equally important to examine productivity and job performance as potential benefits of effective safety and health practices. Strong communication leads to effective teamwork in organizations, essential for ensuring these benefits while keeping workplace hazards in check. Furthermore, AI-driven solutions create new avenues for safety improvement by facilitating real-time reporting, automating danger detection, and fostering stakeholder collaboration. Many industries, including hospitality, have already seen greater efficiency and flexibility from AI adoption [120]. Bringing similar innovations to high-risk environments like coal-fired power plants could strengthen safety protocols, streamline risk management, and create a safer, more productive workplace.

This study provides valuable insights into the function of communication and coordination as proactive safety indicators in high-risk industries, particularly in coal-fired power plants. While existing research has broadly examined safety behaviours, this study advances the field by establishing safety compliance as a key mediator between communication, coordination, and safety outcomes. The findings demonstrate that leading indicators such as communication and coordination do not directly influence safety outcomes but are determined by their effect towards safety behaviours, as argued by Mousavi et al. [121], Reiman & Pietikäinen [119] and Ali et al. [8]. Furthermore, the study offers practical, industry-specific recommendations to enhance safety frameworks, ensuring organizations implement effective communication strategies and coordination mechanisms to mitigate risks. The study’s finding bridges the gap between safety theory and practical application, thus providing meaningful guidance for industry practitioners and policymakers in improving workplace safety and operational resilience.

## Conclusion

This study emphasizes the central role of communication and coordination in promoting workplace safety in coal-fired power plants in Malaysia. The findings show that these practices have a significant impact on safety compliance and participation and serve as essential mechanisms to mitigate occupational risks and improve safety outcomes. Communication and coordination act as proactive measures that promote safety behaviours that reduce negative incidents such as accidents, fatalities, near misses and lost time injuries. The study also highlights the mediating role of safety compliance and emphasizes that the effectiveness of communication and coordination depends on its ability to promote compliance with safety procedures. This finding highlights the need for organizations to prioritize robust safety communication and coordination, which in turn contribute to a culture of safety and operational excellence.

However, the limitations of the study point to opportunities for further research. Future studies should broaden the scope of future studies to include additional safety management practices such as hazard recognition, training, and leadership engagement to develop a more comprehensive safety framework is valuable. Incorporating these domains will provide a more thorough comprehension of workplace safety management. Furthermore, future research could also examine positive outcomes such as increased productivity and worker wellbeing. This will provide a balanced perspective on the benefits, demonstrating how safety initiatives can have holistic benefits for both workers and organizations. Longitudinal studies could provide a more dynamic view of the sustainability of safety procedures and offer insightful information on the long-term effects of coordination and communication on safety results. By addressing these areas, future research can build on the findings of this study to develop holistic strategies to improve safety in the workplace, ensuring not only the reduction of negative outcomes but also the promotion of sustainable organizational performance.

## Supporting information

S1 Table Communication and coordination items in questionnare. (DOCX)

S2 Table Safety behaviours items in questionnare. (DOCX)

S3 Table Safety outcomes items in questionnare. (DOCX)
