# Supplementary material for: Communication and coordination as drivers of safety behaviours and outcomes in coal-fired power plants
Source: PLoS One. 2026 Jan 30;21(1):e0341341. doi: 10.1371/journal.pone.0341341 (PMC12858064; doi:10.1371/journal.pone.0341341)
Supplement: S1 Table — (DOCX) [file pone.0341341.s001.docx]

**S1 Table. List of driven leading indicators use to measured communication and coordination practices.**

| **Construct** | **Label** | **Items** |
| --- | --- | --- |
| **Management Communication** | MNC1 | To what extent are the workplace safety policy’s vision and goals displayed in prominent locations? |
|  | MNC2 | How easy is it to understand and access workplace safety rules and procedures? |
|  | MNC3 | How frequently do workers receive safety performance updates during structured safety meetings? |
|  | MNC4 | How frequently are safety issues discussed in the workplace? |
|  | MNC5 | To what extent does management infrequently or indirectly communicate safety information? |
| **Safety Reporting** | SFR1 | To what extent do workers feel confident that safety issues reported will remain confidential and without retaliation? |
|  | SFR2 | How openly do workers report safety issues to management? |
|  | SFR3 | To what extent do workers trust that management will handle safety reports effectively? |
|  | SFR4 | How effectively do workers receive prompt, helpful, and clear responses to their safety reports? |
|  | SFR5 | To what extent are the outcomes of reported safety issues not shared transparently with workers, including corrective actions? |
| **Supervisory Communication** | SVC1 | To what extent do supervisors actively promote and reward safe behaviours among workers? |
|  | SVC2 | How effectively do supervisors provide constructive feedback on safe and unsafe behaviours? |
|  | SVC3 | To what extent do supervisors and workers collaboratively resolve safety-related issues? |
|  | SVC4 | How clearly does supervisor communication clarify workplace safety responsibilities and procedures? |
|  | SVC5 | To what extent do supervisors fail to consistently support workers in adhering to safety regulations? |
| **OSH Coordination** | SCO1 | How effectively are recurring and outsourced tasks planned with safety considerations in mind? |
|  | SCO2 | To what extent do standardized policies help workers consistently follow safety procedures? |
|  | SCO3 | How effectively do supervisors ensure that tasks are performed safely? |
|  | SCO4 | How systematically does management monitor activities to assess safety effectiveness? |
|  | SCO5 | To what extent do workers not fully understand or take responsibility for following safety regulations? |
