# Supplementary material for: Communication and coordination as drivers of safety behaviours and outcomes in coal-fired power plants
Source: PLoS One. 2026 Jan 30;21(1):e0341341. doi: 10.1371/journal.pone.0341341 (PMC12858064; doi:10.1371/journal.pone.0341341)
Supplement: S2 Table — (DOCX) [file pone.0341341.s002.docx]

**S2 Table. Item used to measure safety knowledge, safety motivation, safety compliance and safety participation.**

| **Construct** | **Label** | **Items** |
| --- | --- | --- |
| **Safety Knowledge** | SFK1 | Do you know how to perform your job safely? |
|  | SFK2 | Do you know how to maintain and improve workplace safety? |
|  | SFK3 | Do you know how to follow safe work procedures? |
|  | SFK4 | Do you know how to reduce workplace accident risks? |
|  | SFK5 | Are you unaware of the hazards and necessary precautions related to your job? |
| **Safety Motivation** | SFM1 | Do you believe workplace safety and health are important? |
|  | SFM2 | Do you prioritize keeping yourself safe at all times? |
|  | SFM3 | Do you actively try to improve workplace safety? |
|  | SFM4 | Do you work to reduce workplace accidents and incidents? |
|  | SFM5 | Do you discourage others from adopting safe practices? |
| **Safety Compliance** | SFC1 | Do you always use the necessary safety equipment while working? |
|  | SFC2 | Do you follow proper safe work procedures? |
|  | SFC3 | Do you ensure the highest safety standards when performing tasks? |
|  | SFC4 | Do you wear personal protective equipment correctly and consistently? |
|  | SFC5 | Have you not reported any workplace injuries, near misses, or hazards in the last 12 months? |
| **Safety Participation** | SFP1 | Do you put effort into improving workplace safety? |
|  | SFP2 | Do you volunteer for safety-related tasks or activities? |
|  | SFP3 | Do you encourage colleagues to work safely? |
|  | SFP4 | Do you address management about workplace safety issues? |
|  | SFP5 | Do you not take action to stop safety violations at work? |
