# Supplementary material for: Communication and coordination as drivers of safety behaviours and outcomes in coal-fired power plants
Source: PLoS One. 2026 Jan 30;21(1):e0341341. doi: 10.1371/journal.pone.0341341 (PMC12858064; doi:10.1371/journal.pone.0341341)
Supplement: S3 Table — (DOCX) [file pone.0341341.s003.docx]

**Table 3.** List of indicators in safety outcomes.

| **Construct** | **Label** | **Items** |
| --- | --- | --- |
| **Occupational Accidents** | OA1 | How often do non-fatal workplace accidents occur annually? |
|  | OA2 | How many accidents result in permanent disabilities? |
|  | OA3 | How many accidents cause temporary disabilities? |
|  | OA4 | How many minor injuries require only first aid? |
|  | OA5 | What is the cost of treating injuries from workplace accidents? |
| **Fatal Accidents** | FA1 | How often do fatal workplace accidents occur? |
|  | FA2 | What are the administrative and legal costs of fatal accidents? |
|  | FA3 | What is the cost of investigating workplace accidents? |
|  | FA4 | How has productivity declined due to losing skilled workers in fatal accidents? |
|  | FA5 | How does replacing workers affect company productivity? |
| **Near Misses** | NM1 | How often do hazardous events without injuries or damage occur? |
|  | NM2 | How often do hazardous events cause minor property damage? |
|  | NM3 | How often are dangerous incidents caused by unsafe worker actions? |
|  | NM4 | How often are dangerous events caused by unsafe conditions? |
|  | NM5 | What is the cost of repairing equipment or facilities after hazardous events? |
| **Lost Time Injury** | LTI1 | How much productive work time is lost due to worker injuries or pain? |
|  | LTI2 | What is the cost of replacement wages or overtime for absent workers? |
|  | LTI3 | How frequently do workers take sick leave? |
|  | LTI4 | How often do operations pause for investigations, impacting productivity? |
|  | LTI5 | What is the cost of lost production due to operational stoppages from accidents? |
